# A dataset for buffering delays due to the interaction between the Nagle algorithm and the delayed acknowledgement algorithm in cyber-physical systems communication

**DOI:** 10.1016/j.dib.2021.107530

**Published:** 2021-10-29

**Authors:** Ahmad T. Al-Hammouri, Rasmus L. Olsen

**Affiliations:** a Department of Network Engineering and Security, Jordan University of Science and Technology, P.O. Box 3030, Irbid 22110, Jordan; b Department of Electronic Systems, Aalborg University, P.O. Box 159, Aalborg 9100, Denmark

**Keywords:** IoT communication, Real-time communication, Sender-side delay, TCP buffering, Protocol parameter tuning, Real-life operating systems

## Abstract

In this article, we provide the research community with a dataset for the buffering delays that data packets experience at the TCP sending side in the realm of Cyber-Physical Systems (CPSs) and IoT. We focus on the buffering that occurs at the sender side due to the the adverse interaction between the Nagle algorithm and the delayed acknowledgement algorithm, which both were originally introduced into TCP to prevent sending out many small-sized packets over the network. These two algorithms are turned on (enabled) by default in most operating systems.

The dataset is collected using four real-life operating systems: Windows, Linux, FreeBSD, and QNX (a real-time operating system). In each scenario, there are three separate different (virtual) machines running various operating systems. One machine, or an end-host, acts a data source, another acts as a data sink, and a third acts a network emulator that introduces artificial propagation delays between the source and the destination.

To measure buffering delay at the sender side, we record for each sent packet the two time instants: when the packet is first generated at the application layer, and when it is actually sent on the physical network. In each case, 10 different *independent* experiment replications/runs are executed.

Here, we provide the full distribution of all delay samples represented by the cumulative distribution function (CDF), which is expressed mathematically by

$$\;\;\;\;\;\;\;\;\;\;\;\;\;\;\;\;\;\;\;\;\;\;\;\;\;F_{X}\left(x\right)=P\left(X\leq x\right)\;,$$

where x is the delay measured in milliseconds, and P is the probability operator.

The data exhibited here gives an impression of the amount and scale of the delay occurring at sender-side in TCP. More importantly, the data can be used to investigate the degree these delays affect the performance of cyber-physical systems and IoT or other real-time applications employing TCP.

## Specifications Table

**Table:** 

Subject	Computer Science
Specific subject area	Computer Networks and Communications, buffering delay in Cyber-Physical Systems and IoT
Type of data	Table
How data were acquired	Server hardware, VMware ESXi Hypervisor, Operating Systems (QNX, Windows, Ubuntu Linux, and FreeBSD Unix), TCP server program, TCP client program, packet sniffing/capturing program, and traffic shaping control program
Data format	Raw, analyzed
Parameters for data collection	Three virtual machines (a source, network emulator, and destination) run atop VMware ESXi hypervisor. The source runs the QNX Neutrino 7.0 real-time operating system. The destination runs one of four operating systems: QNX 7.0, Windows 7, Ubuntu Linux 18.04, or FreeBSD 12.1. The Network Emulator runs Ubuntu Linux 18.04. Each machine is equipped with 4GB of RAM and two CPUs. Each packet exchanged is of size 100B
Description of data collection	The source runs a TCP server program, the destination runs a TCP client program, and the Network Emulator runs the netem utility. The client initiates the TCP connection with the server. Then, the server continuously generates fixed-sized packets spaced evenly in time, and sends them to the client. netem is used to introduce artificial propagation delays between the source and the destination. The buffering delay is obtained by measuring for each packet the two time instants: when the packet is first generated at the application layer, and when it is actually sent at the physical network
Data source location	Institution: Jordan University of Science and Technology
	City/Town/Region: Irbid
	Country: Jordan
	Latitude and longitude (and GPS coordinates) for collected samples/data: 32.4913${}^{\circ }$ N, 35.9875${}^{\circ }$ E
Data accessibility	Repository name: Mendeley Data
	Data identification number: 10.17632/zhbpyvt4g9.1
	Direct URL to data: https://data.mendeley.com/datasets/zhbpyvt4g9/1

## Value of the Data

• The data provide the amount of extra delays that real-time systems suffer when using TCP. The extra delay is due to the buffering that happens at the sender side, which in turn is due to the interaction between the two algorithms: Nagle and Delayed Acknowledgement. These two algorithms are turned on (enabled) by default in most operating systems. The data are obtained for various real-life operating systems and for various network settings.
• Different stakeholders working in the field of CPSs and IoT can benefit from the data, including vendors manufacturing such devices, commercial and open-source software developers developing relevant software packages, committee and working group members working on new supporting protocols and standards, and finally academic and industry researchers investigating CPS and IoT applications.
• These data can be used to study to what degree the buffering delays affect the performance of CPSs and IoT applications. Also, they can be used to investigate different control algorithms to compensate for the delays or to mask the effect of delays.
• We hope these data would stimulate further investigation into the fine-grain tuning of the several configuration parameters of the network protocols at the lower layers (i.e., IP, MAC, and physical layers) to better suite the real-time requirements of CPSs and IoT.

## 1 Data Description

In the main directory, Data, there are **40** sub-directories. Each sub-directory is named in the format: ReceiverOS-NagleAlgorithm-RTTms, where

• ReceiverOS is the operating system running on the receiver machine, and is one of four: QNX Neutrino, Windows, Ubuntu Linux, or FreeBSD,
• NagleAlgorithm is either NagleOff when the Nagle algorithm is disabled, or NagleOn when the Nagle algorithm is enabled, and finally
• RTTms is the round-trip propagation delay introduced between the sender and the receiver machines, in milliseconds. It is one of five values: 10 ms, 30 ms, 100 ms, 500 ms, and 1000 ms.

For example, the directory Windows-NagleOff-30ms is for the Windows operating system when the Nagle algorithm is disabled and when the round-trip propagation delay between the source and the destination is 30 ms.

Each sub-directory contains **42** data files in ASCII format as follows:

• Sender-out-i.txt, where i=0,1,2,⋯,9 (10 files). Each file corresponds to a single run/replication, and gives the timestamp (in seconds) when the application layer generated the given packet. The format of the file is as follows
  ID  Ts
  where ID is the packet number (starting at 1), and Ts is the timestamp.
• Sniffer-i.txt, where i=0,1,2,⋯,9 (10 files). Each file corresponds to a single run/replication. The format of the file is as follows
  Ts  SrcIPAddr.SrcPortNo  DstIPAddr.DstPortNo  [Flags]  length  yyy
  where
  - Ts is the timestamp (in seconds) when a TCP segment is sent out on the wire,
  - SrcIPAddr, SrcPortNo, DstIPAddr, DstPortNo are the source IP address, source port number, destination IP address, and destination port number, respectively,
  - Flags is the TCP flags, A, F, S, P, U, etc., and
  - yyy is the length/size of the TCP segment being sent in bytes.
• SnifferTimestamp-i.txt, where i=0,1,2,⋯,9 (10 files). Each file corresponds to a single Sniffer-i.txt file. For example, SnifferTimestamp-5.txt corresponds to Sniffer-5.txt. Whereas each Sniffer-i.txt file gives the timestamp when each TCP segment is sent out on the wire, the file SnifferTimestamp-i.txt gives the timestamp when each application-level message/packet is sent out on the wire. Therefore, SnifferTimestamp-i.txt is an expansion of Sniffer-i.txt because several application-layer packets may get sent out in the same TCP segment. The format of the file is as follows
  Ts  Len
  where Ts is the timestamp when the application-level packet is sent on the network, and Len is the size of the packet in bytes.
• App-Net-Delay-Sniffer-i.txt, where i=0,1,2,⋯,9 (10 files). The format of the file is as follows
  ID  Ts  Ts  Len  Delay
  where
  - The first two columns in each App-Net-Delay-Sniffer-i.txt are the same two columns in the corresponding Sender-out-i.txt file,
  - The third and the fourth columns in each App-Net-Delay-Sniffer-i.txt are the same two columns in the corresponding SnifferTimestamp-i.txt file, and
  - The last column (Delay) in each App-Net-Delay-Sniffer-i.txt file is the **buffering delay** at the sender side measured in microseconds. That is, each entry in the fifth column, i.e., Delay, is the difference between the entry in the same row of the third column and the entry in the same row of the second column in the same App-Net-Delay-Sniffer-i.txt file, converted into microseconds.
• App-Net-Delay-ALL.txt (one file). The file combines all delay samples from all runs/replications in a single file. The format of the file is as follows
  ID  Delay
  where ID is the same ID from each Sender-out-i.txt file, and Delay is the corresponding buffering delay at the sender side, in microseconds.
• CDF.txt (one file). The file gives the distribution of all delay samples across all runs in terms of the cumulative distribution function (CDF), which is expressed mathematically by
  $$F_{X}\left(x\right)=P\left(X\leq x\right)\;,$$
  where x is the delay measured in milliseconds, and P is the probability operator.
  The format of the file is as follows
  Delay  CDF
  where Delay is the buffering delay measured in milliseconds, and CDF is the CDF value.

**Fig. 1:**
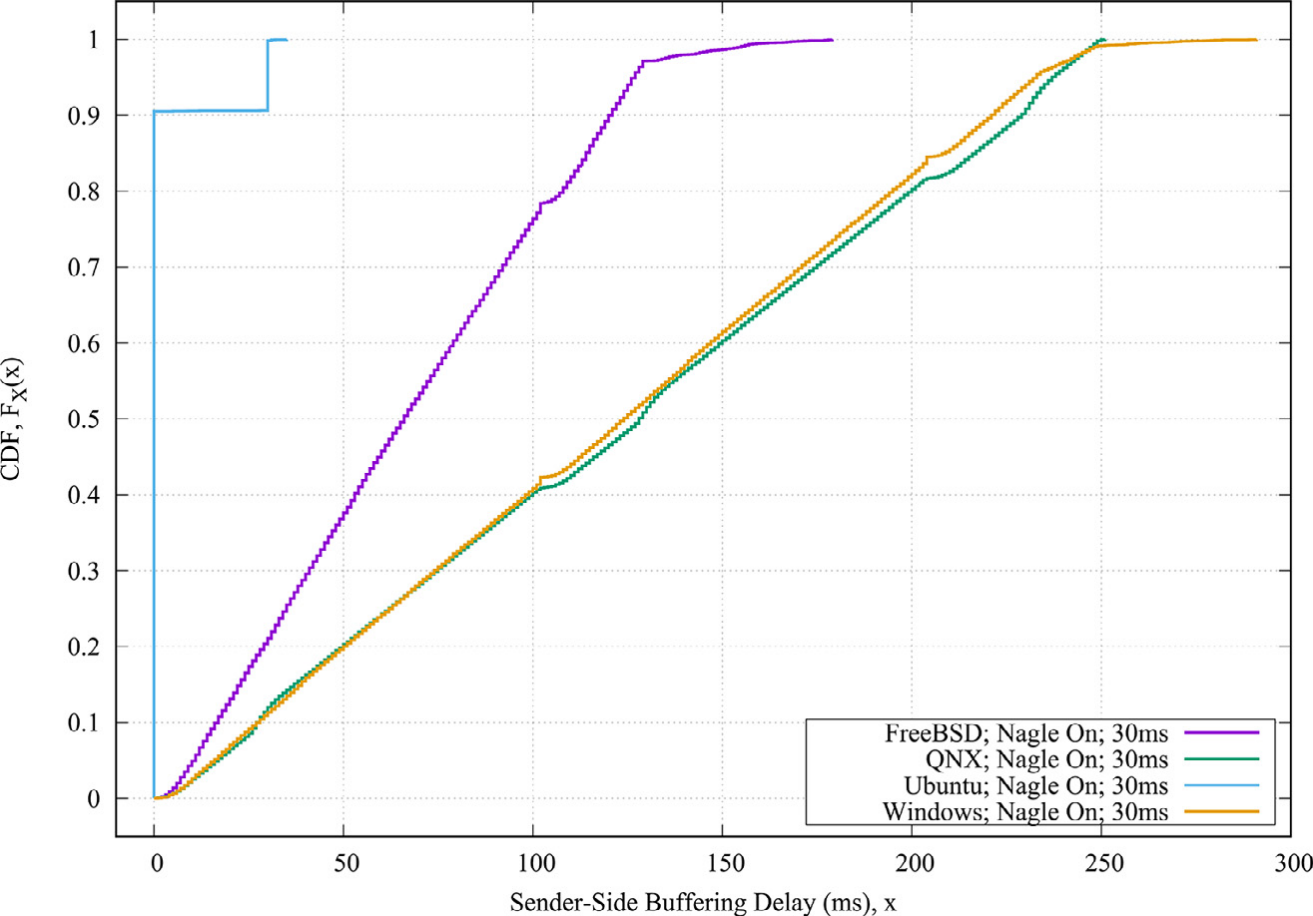
The CDF values of the sender-side buffering delays for the four different operating systems when the Nagle algorithm is enabled and when the round-trip propagation delay between the sender and the receiver is 30 ms.

For example,

• Fig. 1 shows the CDF plots of the four different operating systems when the Nagle algorithm is enabled and when the round-trip propagation delay between the sender and the receiver is 30 ms.

**Fig. 2:**
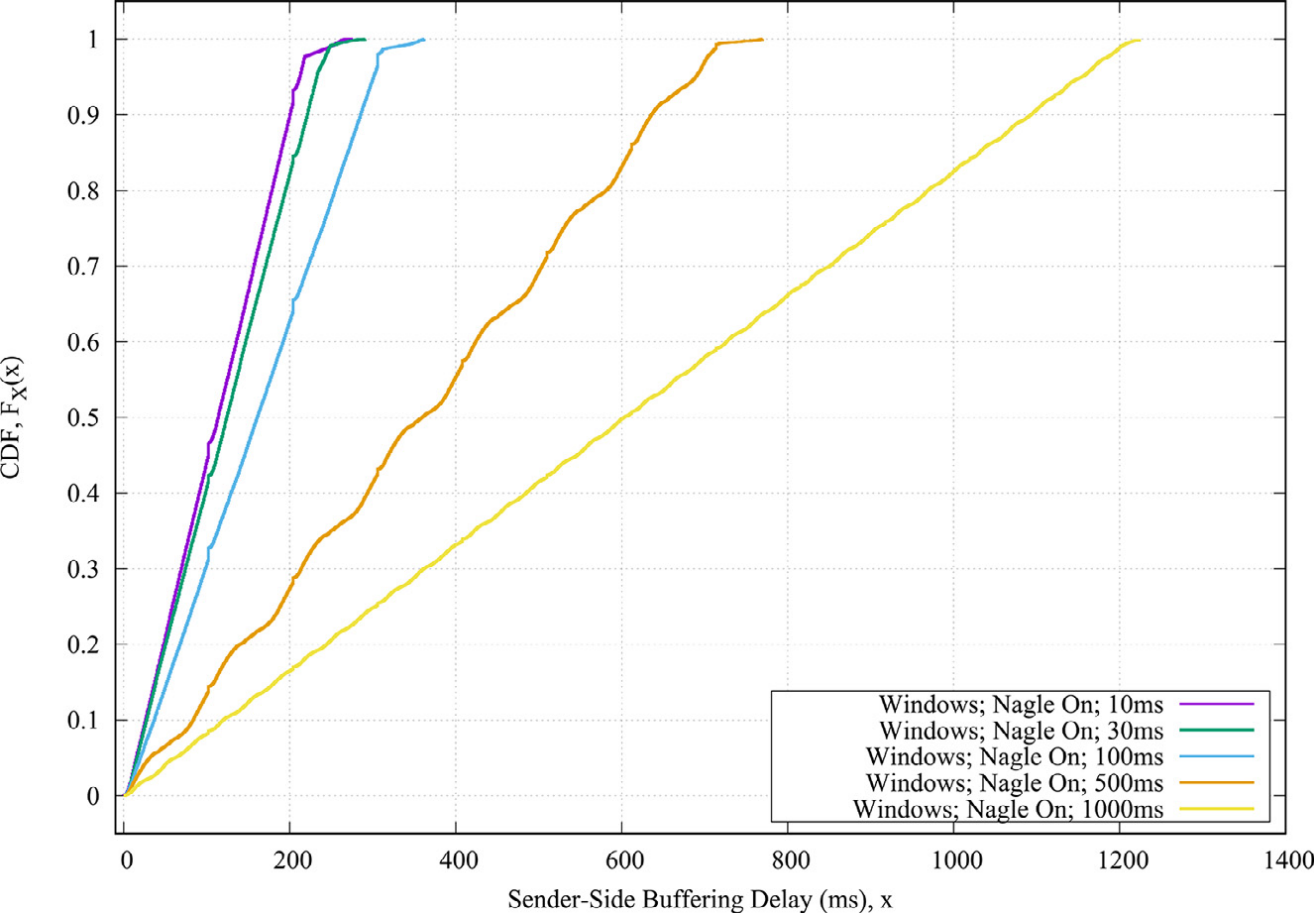
The CDF values of the sender-side buffering delays for the Windows operating systems when the Nagle algorithm is enabled and for different round-trip propagation delay between the sender and the receiver.

**Fig. 3:**
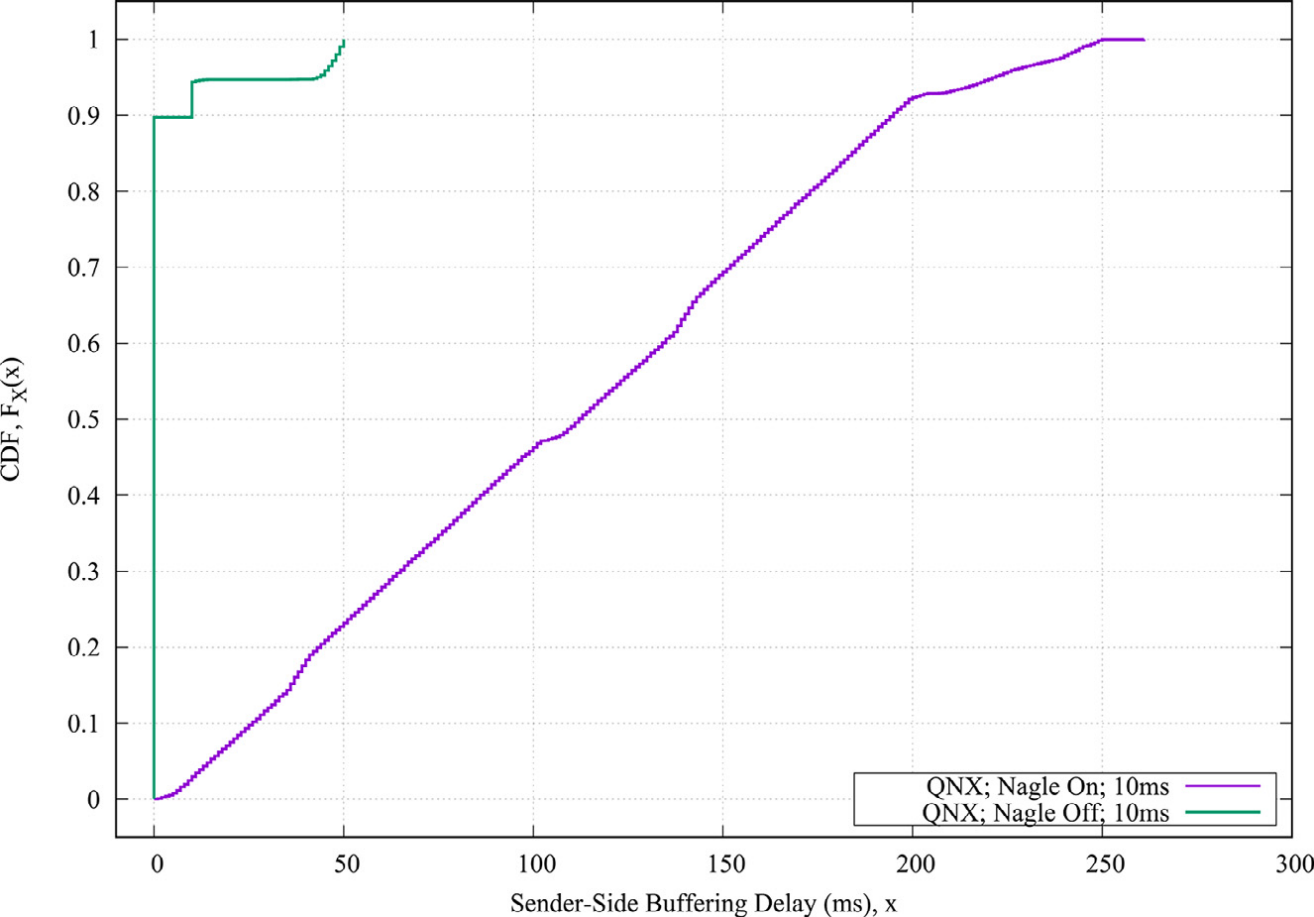
The CDF values of the sender-side buffering delays for the QNX operating systems when the Nagle algorithm is both enabled and disabled, and when the round-trip propagation delay between the sender and the receiver is 10 ms.
• Fig. 2 shows the CDF plots of the Windows operating systems when the Nagle algorithm is enabled and for different round-trip propagation delay between the sender and the receiver.
• Fig. 3 shows the CDF plots of the QNX operating systems when the Nagle algorithm is both enabled and disabled, and when the round-trip propagation delay between the sender and the receiver is 10 msec.

## 2 Experimental Design, Materials and Methods

Here, we preset the experimental environment that we used to collect the buffering delay at the sender side in various real-life operating systems.

**Fig. 4:**
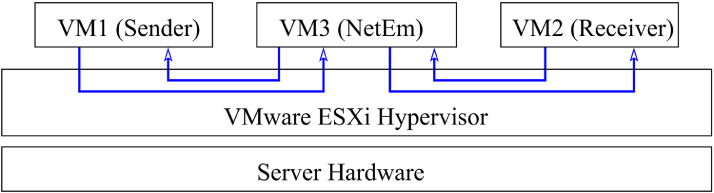
Experimental Setup: VM1, VM2, and VM3 are virtual machines running on the physical machine and are connected via a software router inside the hypervisor.

The setup comprises three *virtual* machines running on the same virtualization software, which is VMware ESXi hypervisor. The VMware ESXi hypervisor runs over a server hardware. One of the virtual machines is the data source (sender); another is the destination (receiver); and the third is a network emulator (NetEm). Fig. 4 depicts the experimental setup.

Logically, the source that sends data in a CPSs is usually an embedded device (a specialized hardware and a specialized software). Examples for data sources include Plant Control in a SCADA system and Phasor Measurement Units (PMUs) in power systems. Since embedded devices run a real-time operating system, we choose for the virtual machine VM1 the QNX Neutrino 7.0 real-time operating system [1]. The QNX operating system is a highly dependable, industry-grade operating system that is utilized in more than 175 million cars worldwide [2], and in different industrial Programmable Logic Controllers (PLCs) and control systems [3].

On the other hand, the data destination or sink can be an embedded device, or it can also be offered as a software package to be installed on a computer system, e.g., a regular PC. In the latter case, the software package is installed on commonly used operating systems, such as Windows, Linux, and Mac OS; see for example [4], [5], [6]. Examples of data destinations include Dispatch units in a SCADA system and Phasor Data Concentrators (PDCs) in power systems. Therefore, for VM2, we choose to experiment with four operating systems: QNX Neutrino 7.0, Windows 7, Ubuntu Linux 18.04, and FreeBSD 12.1 Unix (macOS borrows heavily from FreeBSD [7]).

The source runs a TCP server program while the destination runs a TCP client program. The client initiates the TCP connection with the server. Then, the server continuously generates fixed-sized packets spaced evenly in time, and sends them to the client. Each packet exchanged is of size 100B and the interval between the generated data packets is 100 ms.

Finally, VM3 runs Ubuntu Linux 18.04 and is equipped with the netem (Network Emulator) utility [8], which is used to emulate different network conditions by introducing artificial propagation delays between the source and the destination.

**Fig. 5:**
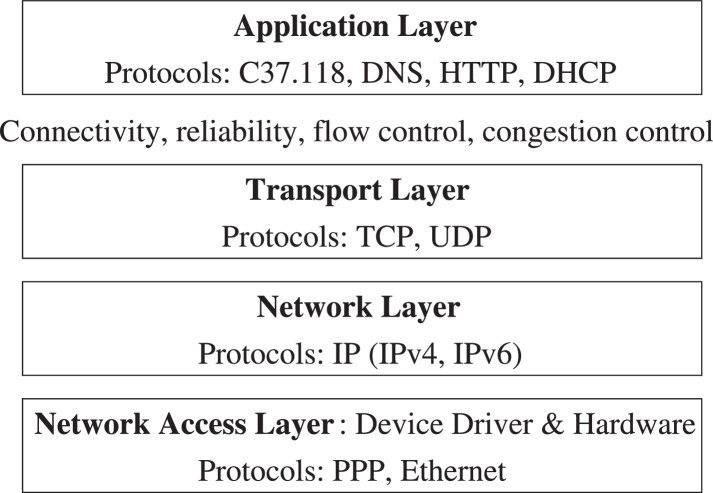
The Layering Model of Network Protocols with example protocols in each layer.

To measure extra buffering delay at the sender side, we record for each packet the two time instants: when the packet is first generated at the application layer, and when it is actually sent at the physical network. The first time instant is obtained by invoking the clock_gettime system call just before the sender process writes the data to the TCP socket. The second time instant is obtained via a packet capturing/sniffing program. The difference between the two time instants gives the time that each packet incurs at the transport layer (i.e., the time each packet is held in the TCP send buffer before being forwarded); see Fig. 5.

Finally, to eliminate the random noise and the inherent stochastic variability in the output data, 10 different *independent* experiment replications/runs are executed for each case. Each run lasts until 1005 packets are successfully sent out.

We emphasize that the used experimental setup is sufficient to the studied facets of TCP because we are not studying any interaction and contention between traffic of different sources; rather, we are studying the delay caused by the buffering of packets at sender side. This buffering is due only to the mechanisms implemented by the end nodes themselves. As such, there is no need to involve more nodes (e.g., more senders, and routers or switches), more links, or cross (background) traffic.

## Ethics Statement

This work involved **no** human subjects and **no** animal experiments.

## CRediT authorship contribution statement

**Ahmad T. Al-Hammouri:** Conceptualization, Methodology, Software, Validation, Investigation, Data curation, Writing – original draft. **Rasmus L. Olsen:** Conceptualization, Writing – review & editing.

## Declaration of Competing Interest

The authors declare that they have no known competing financial interests or personal relationships which have, or could be perceived to have, influenced the work reported in this article.
